# Comment on: “Artificial Intelligence and Ophthalmology”

**DOI:** 10.4274/tjo.galenos.2020.98354

**Published:** 2020-12-29

**Authors:** Thiago Gonçalves Dos Santos Martins

**Affiliations:** 1 Federal University of São Paulo, Department of Ophthalmology, Brazil

**Keywords:** Artificial intelligence, machine learning, deep learning, ophthalmology, medical ethics

## To the Editor

In response to the article titled “Artificial Intelligence and Ophthalmology” published in your esteemed journal, which is a well thought of and written paper, I would like to raise a few points regarding this study. The article discusses developments and potential practices regarding the use of artificial intelligence in the field of ophthalmology, and the related topic of medical ethics.^1^

The development of artificial intelligence algorithms requires a large number of ophthalmic images to be developed. The effectiveness of the algorithm after being developed needs to be validated in clinical trials with a different database than the one used for training the algorithms, thereby evaluating the reliability and efficiency of the algorithm. Bearing in mind that the standard of effectiveness of the algorithms varies between studies, it is difficult to compare algorithms with each other.^2^ Regarding ethical aspects, it is important that patient privacy rules are respected when sharing data between the research centers that develop the algorithms, which must generate the data anonymously.

Diseases such as diabetic retinopathy, macular degeneration, glaucoma, and retinopathy of prematurity are prevalent diseases that have a large amount of data stored in large study centers. However, rare eye diseases such as retinal dystrophies have greater difficulty in creating artificial intelligence algorithms, as they do not have much stored data.

The creation of algorithms can reduce diagnostic errors and facilitate the monitoring of ophthalmological diseases in regions that do not have an adequate number of ophthalmologists.^3^

Concerning ethical aspects, the bias in data collection can affect the generalization of the model trained for use in the population. Studies in different populations can minimize these problems.

In this way, algorithms have the potential to perform numerous tasks more quickly and efficiently than humans, such as data and information processing. However, they have limitations, such as the lack of perception of the social and psychological aspects of human nature that can eventually influence the diagnosis.
